# Antibody Profiling: Kinetics with Native Biomarkers for Diagnostic Assay and Drug Developments

**DOI:** 10.3390/bios13121030

**Published:** 2023-12-14

**Authors:** Ute Jucknischke, Sebastian Friebe, Markus Rehle, Laura Quast, Sven H. Schmidt

**Affiliations:** Diagnostic Solutions, Reagent Research and Design, Department Antibody and Protein Technologies, (DSRRA) at Roche Diagnostics GmbH, Nonnenwald 2, 82377 Penzberg, Germany

**Keywords:** surface plasmon resonance, SPR, Biacore, native biomarker, neurofilament light chain, NFL, GDF15, interaction kinetics

## Abstract

Despite remarkable progress in applied Surface Plasmon Resonance (SPR)-based methods, concise monitoring of kinetic properties for native biomarkers from patient samples is still lacking. Not only are low concentrations of native targets in patient samples, often in the pM range, a limiting and challenging factor, but body fluids as complex matrices furthermore complicate measurements. The here-described method enables the determination of kinetic constants and resulting affinities for native antigens from patients’ cerebrospinal fluid (CSF) and sera binding to antibodies. Using a significantly extended target-enrichment step, we modified a common sandwich-assay protocol, based on a primary and secondary antibody. We successfully analyze antibody kinetics of native targets from a variety of origins, with consistent results, independent of their source. Moreover, native neurofilament light chain (NFL) was investigated as an exemplary biomarker. Obtained data reveal antibodies recognizing recombinant NFL with high affinities, while showing no, or only significantly weakened binding to native NFL. The indicated differences for recombinant vs. native material demonstrate another beneficial application. Our assay is highly suitable for gaining valuable insights into characteristics of native biomarkers, thus impacting on the binder development of diagnostic reagents or pharmaceutical drugs.

## 1. Introduction

The development of pharmaceutical drugs as well as diagnostic assays for a native target requires fine-tuned kinetic properties of the drug or detection molecule to result in the desired effect or readout. Many if not most state-of-the art diagnostic assays are based on antibodies binding their target molecule [1,2,3,4,5]. Identifying those high-affine and highly specific antibodies is challenging as recombinant expressed target molecules can behave differently compared to their native counterparts. Post-translational modifications, unknown isoforms, affinity-tags for purification or overall protein/target quality differences are only some of the hurdles to overcome in the antibody selection process. Besides the differences of recombinant and native proteins, the complex background given for samples from blood, sera, cerebrospinal fluid (CSF) or similar matrices compared to standard buffers, like HEPES or PBS, can have a tremendous impact on the target recognition as well.

Growth differentiation factor 15 (GDF15) is, for example, measured in serum samples. The physiological concentration of healthy controls is around 450 pg/mL while under malignant cancer conditions it is elevated up to 10,000 pg/mL or even up to 100,000 pg/mL. The protein which belongs to the transforming growth factor beta (TGFβ) superfamily is known for functioning as a hormone, stress-induced cytokine or stress-sensitive circulating factor. Moreover, in cancer the dimeric protein plays a role in metabolic diseases, inflammation and cardiovascular diseases like hypertrophy and heart failure [6].

Another example measured in CSF or blood samples—serum and plasma alike—is the scaffold protein neurofilament light chain (NFL). It is used as a biomarker for axonal degeneration, and is therefore of great interest for many neurodegenerative diseases like Alzheimer’s disease (AD), atypical forms of parkinsonian syndromes, fronto-temporal dementia (FTD), amyotrophic lateral sclerosis (ALS), multiple sclerosis (MS) and many more, as well as traumatic brain injuries, stroke and neurological damages in oncology [7,8,9]. The release of NFL to CSF or blood is significantly increased after damaging or degenerating processes affecting neuronal axons [10]. As the physiological, as well as disease-associated concentrations in CSF or blood samples are in the pg/mL-range, there is a need for highly sensitive state-of-the-art diagnostic assays, which depend on highly specific and affine antibodies.

Therefore, it is of great importance to test for native sample recognition and possible changed interaction kinetics as early as possible in the antibody selection process. To achieve this goal, an increasing number of surface plasmon resonance (SPR)-based assay setups can be found in the literature [11]. However, the focus of those assays is most often based on the detection and concentration determination of target molecules in native samples like serum or CSF samples. All of those assay setups do not address the determination of kinetic rate constants.

For example, Xia et al. showed an SPR approach to the detection of amyloid-β peptides in a two-digit pM range in CSF samples [12]. They used a capture antibody on the SPR surface to bind amyloid-β peptides, and in a second step injected a preformed complex of a detection antibody and streptavidin, thereby achieving a signal amplification in the SPR sensor. Based on their approach, they were also able to determine apparent affinities/avidities by applying the Langmuir isotherm, an equilibrium binding model, to their data set. The latter determination, however, was carried out with purified peptides and not with CSF samples. In addition, by only determining the affinity/avidity, one loses important information of the underlying interaction kinetics, which are important for the diagnostic assay or drug development.

Other examples in the literature were mostly focusing on the signal amplification, which was more sensitive for the detection of lower marker concentrations, and which reached down to a remarkable detection limit in the aM-range [13,14,15,16,17]. Although this shows how sensitive SPR approaches can be in determining low-abundant markers in body fluids, no kinetic comparisons between native and recombinant proteins were made, although antibody detection can differ greatly between these sources. This becomes of special interest, as most antibodies are generated against the recombinant rather than native protein. 

Especially for diagnostic assay and drug developments, the kinetic rate constants are important for deciding on the best in class antibodies. While for diagnostic assays the time to equilibrium is an important criteria for most high-throughput, automated in vitro diagnostic platforms, binding characteristics for drugs need to be fine-tuned to achieve the desired pharmacokinetics. To determine those kinetic values for native targets, Single Cycle Kinetics, introduced to the SPR field by Robert Carlson et al., were chosen by us to solve this issue [18]. His approach allows the full kinetic characterization of an antibody–target interaction in a single cycle. By combining this approach with a sandwich setup and the use of the corresponding Fab of the antibody of interest, we were able to enrich the native target on the surface and discard potential interfering biological fluid components before the actual interaction analysis. In addition, we were also able to use as little of the precious native sample as possible, while at the same time determining all kinetic interaction constants. This allows us for the first time to compare the kinetic performance of antibodies between the recombinant and native target. Therefore, this assay can also help to identify structural differences between native targets and recombinant calibrators. 

Here, we determined the affinities and kinetic rate constants of antibodies binding to different low-abundant native biomarkers like NFL in CSF, GDF15 in serum or sTREM-1 in plasma samples, and their recombinant counterparts. 

## 2. Material and Methods

### 2.1. SPR Instruments and Software

The SPR experiments were performed on GE Healthcare BIAcore™ T200 and 8K (+) instruments with BIAcore™ T200 Control SW V2.0.2 and Evaluation SW 3.2, and 8K Control-SW V3.0.11.15423 or V4.0.8.20368 and Insight Evaluation SW V3.0.11.15423 or V4.0.8.20368. 

For the simulation of the target-enrichment time, the SW Trace Drawer 1.8.1 from Ridgeview instruments AB was used.

### 2.2. Consumables, Sensor Chips, Reagents and Buffers

Standard sensor CM5-chips Series S and amine coupling kits from Cytiva were used. As system buffers, HBS-N pH 7.4, containing 10 mM HEPES, pH 7.4, and 150 mM NaCl, and HBS-EP+, pH 7.4, containing 10 mM HEPES, pH 7.4, 150 mM NaCl, 3 mM EDTA and 0.05% (*w*/*v*) Tween20 were used, all from Cytiva. PBS-Buffer 10× (0.2 M phosphate buffer with 27 mM KCl, 1.37 M NaCl) from Roche was used, diluted 1: 10 with 0.5% Surfactant Tween 20 (Sigma-Aldrich, St. Louis, MO, USA), as additive. 

The respective system buffer was supplemented with 1 mg/mL CMD (Carboxymethyldextran, Fluka, Charlotte, NC, USA) and used as sample buffer for the sample dilution. Antibodies from Jackson ImmunoResearch were used as capture systems: (I) Polyclonal goat anti-rabbit IgG Fc capture antibody, GARbFcγ, Code-No. 111-005-046, (II) Polyclonal rabbit anti-mouse IgG Fc capture antibody, PAK<M-IgG(Fcy)>K, Code-No. 315-005-046, (III) Polyclonal goat anti-mouse IgG Fc capture antibody PAK<M-IgG(Fcy)>Z, Code-No. 115-005-071, or (IV) Polyclonal rabbit anti-mouse F(ab’)_2_ capture antibody, PAK<M-IgG F(ab’)2>K, Code-No. 315-005-047.

### 2.3. Antigens

Recombinant human GDF15 (aa Ala197-Ile308, Accession # Q99988, N-terminal His & Asn199), CHO-derived, mass 13 kDa (monomer), disulfide-linked homodimer from R&D-Systems, Product No. Catalog #: 957-GD, Lot# EHF708102; purified recombinant human neurofilament light core domain (NFL core) (aa93-396), expressed in E. coli, Roche in-house, mass 36.5 kDa (monomer), Gaetani et al., 2018 [19]; and purified recombinant soluble, extracellular domain of human TREM1 Protein, residues 1 to 205 of SW: TREM1_HUMAN, Q9NP99, fused to a C-terminal His-Tag, HEK- derived material, Roche in-house, mass 22.5 kDa.

The patient-derived human sera, with the native biomarkers GDF15 “healthy donors” are samples collected with informed consent, all anonymized. The study was conducted in compliance with the FDA Code of Federal regulations (21CFR 50 and 56), and approved by the Diagnostics Investigational Review Board. 

Patient consent for patient-derived samples regarding growth differentiation factor 15 (GDF15) of origin “oncological disease”, “cardiovascular” and “pregnant donors” was waived, due to usage of leftover samples from clinical routine: Here, 3 leftover serum samples of three anonymized subjects from clinical routine are used for GDF15 derived from patients with oncological disease, 3 leftover serum samples of three anonymized subjects from clinical routine are used for GDF15 derived from “cardiovascular origin” and 3 leftover serum samples of three anonymized subjects from clinical routine are used for GDF15 derived from “pregnant donors”. Patient consent was waived, due to usage of leftover samples from clinical routine for the patient-derived sTREM-1 samples “Intended Use” and “LPS-stimulated”: the analyzed sTREM-1 samples “Intended Use” represent pooled plasma samples that were collected as leftover samples of anonymized subjects from clinical routine. The analyzed “LPS-stimulated” sample represents pooled plasma samples, stimulated with LPS (Sigma-Aldrich) c = 0.5–5.0 µg/mL for 24 h at 35 °C; all were anonymized.

Patient consent for patient-derived samples regarding neurofilament light chain (NFL) was waived due to usage of leftover samples from clinical routine. For data regarding neurofilament light chain (NFL), two leftover cerebrospinal fluid samples of two anonymized subjects from clinical routine were used.

### 2.4. Antibodies

Rabbit-derived monoclonal Anti-NFL-antibodies IgG-format and Fabs were used, generated and purified by Roche in-house; commercial mouse-derived monoclonal Anti-GDF15-antibodies from R&D-Systems, mouse-derived monoclonal Anti-sTREM-1-antibodies IgG-format and Fabs were used, generated and purified by Roche in-house; and non-target-related monoclonal rabbit antibody K-N-IgG (Roche in-house) or non-target-related monoclonal mouse antibody (MAK<CK-MM>M-33-IgG, Roche in-house) was used for referencing. Optionally, the identical antibodies served as blocking reagents to saturate free binding sites of the capture system subsequently to the capturing of the primary antibody.

### 2.5. Functionalizing the Sensor

A rabbit- or mouse-antibody capture system was immobilized on a standard sensor surface CM5 at 25 °C, using HBS-N pH 7.4 as a system buffer. The polyclonal antibodies, serving as capture system, were amine coupled using the EDC/NHS-chemistry, according to the manufacturer’s instructions. Ligand densities between 9000 RU and 15,000 RU were achieved. After each measurement cycle for the interaction analysis, the capture systems were regenerated at 20 µL/min by subsequent injections with acidic regeneration solutions, optimized for each capture system, with 10 mM Glycine buffers of varying pH 1.7–2.25 or 100 mM HCl.

### 2.6. Interaction Analysis via SPR

The interaction analysis includes three main steps: the capturing of the primary antibody by a CM5 sensor chip surface-displayed capture system, the target enrichment of the native marker protein provided in a complex biological fluid, and finally, the monitoring of the binding properties of the second antibody binding to the surface-displayed, native target. For those three steps, we defined following nomenclature:

(1) Capturing.

(2) Target Enrichment. 

(3) Binding-kinetics of the 2nd binder. 

After each measurement cycle for the interaction analysis, the capture systems were regenerated, as stated in Section 2.5.

The usual referencing was applied: on each of the 8 channels of the 8K instrument, flow cell 1 (Fc1) served as reference, and was subtracted from the measuring Fc2. 

For the T200 instrument, Fc1 served as reference, and was subtracted from the measuring Fc2. 

A sandwich-based assay format was used, where the low-concentrated target is enriched via binding to a captured, surface-displayed primary antibody. Capturing of the primary antibody is performed via the above-stated capture systems. The achieved capture levels (CL) are monitored. 

(1) The primary antibody, with concentrations up to 300 nM, is captured via injections for 1–2 min at a flow rate of 5 or 10 µL/min on Fc2. A non-target-related antibody of the same species as the primary antibody, a rabbit antibody K-N-IgG and a non-target- related mouse antibody (MAK<CK-MM>M-33-IgG), respectively, was used as a control on the reference Fc1. 

(2) Next, the native target provided in a given biological fluid, diluted in the appropriate running buffer to approx. 10–50 pM, is injected via consecutive injections for a total time of up to 140 min, at flow rate of 5 or 10 µL/min. In the case where the concentration of the native target is below 20 pM, the biomarker is injected non-diluted, as stated above.

For comparing the native vs. recombinant target, instead of the native target, the recombinant target is used, as stated above.

(3) Finally, an antibody Fab Fragment of the second antibody is injected. 

Using the single-cycle kinetics, a series of five increasing antibody concentrations is injected. As sample preparation, the antibody concentration series is diluted in the sample buffer, using a dilution factor of 3, starting from the highest concentration c1. The optimal concentration range depends on the individual affinity of the analyzed interaction. 

The association phase is monitored for 3 or 5 min, and the dissociation phase is monitored for 30 and 60 min, at a flow rate of 30 µL/min.

A plain sample buffer with concentration zero for each second antibody sample served as blank for the double referencing.

### 2.7. Blocking 

In case of the IgG format of the 2nd binder being of the same species as the primary antibody, free binding sites of the capturing antibody need to be blocked on Fc1 and 2, subsequently to the capturing of the primary antibody. The blocking was performed with stated non-target related antibodies, also used for referencing on Fc1. The injection of the blocking antibodies, concentration 1 mM, was applied to Fc1 and 2 for 3 min, at a flow rate of 30 µL/min, subsequently to the primary antibody capturing. 

### 2.8. Comparing and Ranking the Epitope Accessibility of Antibody Pairs

Alternatively, the ranking of certain antibody pairs regarding epitope accessibility could be investigated using the described assay format. Here, only a single concentration of the second antibody is sufficient, instead of using the single-cycle kinetics. All other steps are applied as stated in Section 2.6.

The response of the second antibody binding to the target is used as a read-out and quantification of the epitope accessibility of the respective analyzed antibody pair.

### 2.9. Evaluating the SPR Data

Single-cycle kinetics were evaluated using the vendor’s SW. The kinetic constants were evaluated using a Langmuir 1:1 fitting model, RI = 0, Rmax global. The ratio of the rate constants determines the dissociation equilibrium constants *K_D_* [M], known as affinity for monovalent binding.

*K_D_* = *k_d_/k_a_* (1)

In cases where an antibody IgG format is used as a second antibody, the measured dissociation equilibrium constant represents avidity rather than affinity, due to possible bivalent binding.

The antibody/antigen complex half-life was calculated in minutes, according to the formula

*t_/2diss_* = *ln*(2)/(*k_d_* × 60) (2)

## 3. Theory and Calculations

### 3.1. Sandwich-Assay Setup

We envisioned investigating the functionality of binders and their potential as reagents in diagnostics’ immuno-based assays or as pharmaceutical drugs using native targets from patient samples early on in the SPR-based development process. Furthermore, we seek to overcome the challenges of measuring kinetic properties in the presence of complex matrices by using the native target as ligand—surface-displayed and enriched via a primary antibody. 

**Figure 1:**
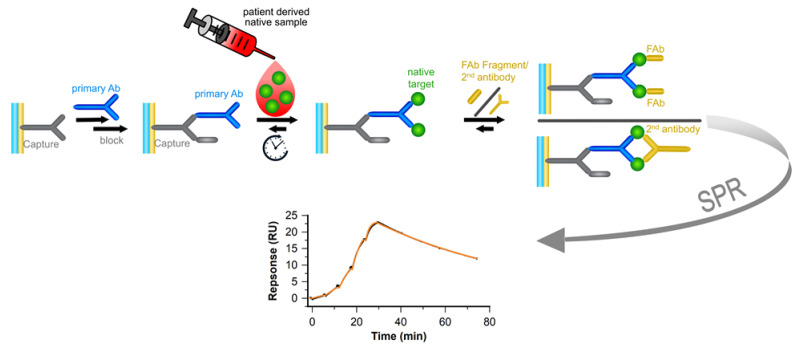
An SPR-based sandwich-assay approach is used to determine the kinetic binding constants of a Fab Fragment/2nd antibody binding to native biomarkers. The only prerequisite is an antibody pair, which forms a sandwich complex with the target of interest, but does not compete for the same target epitope. In that manner, the native or recombinant target is presented via the primary antibody, free to bind the second binder. The native target is injected to a capture system-bound primary antibody for a prolonged time, up to 80–140 min. Then, the second binder, either a monovalent binding Fab or a divalent IgG, is injected. Here, five consecutive injections of the second binder with increasing concentrations are applied. Using this so-called single-cycle kinetics, the kinetic rate constants *k_a_* and *k_d_* and resulting affinity (Fab)/avidity (IgG) of the 2nd binder binding to the native target are determinable.

Our method is based on antibody pairs, which form a sandwich complex with the antigen but do not compete for the same antigen epitope. In that manner, the native antigen is presented via the first antibody with the orientation which is free to bind the second antibody. The binding kinetics of the second antibody is measured with subsequent injections of increasing concentrations. These so-called single-cycle kinetics were originally described by Karlsson et al. [18]. To obtain the affinity rather than avidity, an antibody Fab-Fragment is used as the 2nd binder, ensuring monovalent binding (see Figure 1).

If the second antibody is of the same species as the primary antibody, an additional blocking step is needed for Fc1 and 2 (see Section 2.7): to saturate free-capture-system binding sites, the above described non-target-related antibody is injected, subsequent to the capturing of the primary antibody. 

In the case where the assay read-out is aiming for the comparison and ranking of a selection of certain antibody combinations, the 2nd binder is applied with a single concentration. Here, the epitope accessibility of the native target for certain pairs of antibodies could be quantified and ranked. 

Furthermore, antibodies not binding the native target could be easily identified, due to an abolished binding of the native target.

In the following examples, we use the descriptive nomenclature for the three main steps taking place subsequently, in each measuring cycle, to easily distinguish between the steps:

(1) Capturing
(2) Target-Enrichment
(3) Binding-kinetics of the 2nd binder

### 3.2. Target-Enrichment Step

The enrichment of a native target via a surface-displayed, primary antibody to a sufficient density, enables the subsequent kinetic characterization of a second binder. Several parameters affect the target enrichment, i.e., the density of the covalently bound capture system, the density of the captured primary antibody, the concentration of the target, the time for the target enrichment and, finally, the kinetic properties of the target binding to the primary antibody.

The low-abundant native antigen, often only of low concentration in the low picomolar range, will be compensated by an extended injection time of the target, up to 1–3 h. Superior kinetic properties and high density of the primary antibody with fastest complex formation and sufficient complex stability will shorten the needed enrichment time. Furthermore, the biochemical characteristics of the native target can impact the enrichment time, as well. Possible bi- or multivalent binding of a dimeric or oligomeric native target to the primary antibody can result in a pseudo-stabilized primary antibody/target complex, and would shorten the needed enrichment time.

### 3.3. Simulating the Targets’ Enrichment Time 

**Figure 2:**
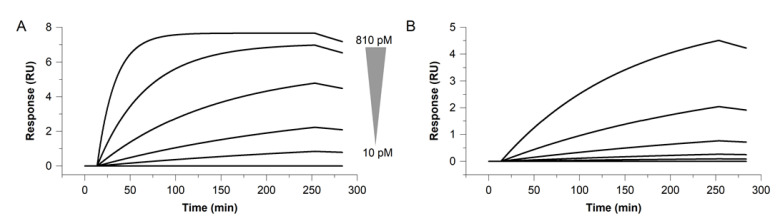
Simulation for low concentrated native target binding to surface-displayed primary antibody. Simulated data for a series of decreasing native-target concentrations of c = 810, 270, 90, 30 and 10 pM binding to a surface-displayed primary antibody. Simulation for a fast association rate constant *k_a_* = 1.0 × 10^6^ M^−1^ s^−1^ (**A**) vs. a factor-10 lower association rate constant of *k_a_* = 1.0 × 10^5^ M^−1^ s^−1^ (**B**). A dissociation rate constant of *k_d_* = 3.6 × 10^−5^ s^−1^ was used for both simulations (**A**,**B**).

We simulated the interaction for primary antibodies binding to a target, based on the considerations described in Section 3.2. In order to determine necessary enrichment times, calculations were performed with set kinetic constants and target concentrations (see Figure 2), using equations described by Karlsson et al. [20]. It is essential that primary antibodies form a sufficiently stable antibody–target complex with monomeric targets, which is why the primary antibodies used in our proposed assay setup exhibited complex half-life times of at least *t_/2diss_* = 60 min. Additionally, these primary antibodies possess superior fast complex-formation velocities, binding the target with an association rate constant of *k_a_* > 1.0 × 10^6^ M^−1^ s^−1^. The simulation clearly shows that kinetic properties of the primary antibody, as well as the target concentration, can have a significant impact on the surface enrichment (see Figure 2).

The simulation shows that a native biomarker at a theoretical concentration of c = 810 pM reaches equilibrium after approx. 120 min when binding to the surface-displayed primary antibody of superior fast complex-formation velocity, whereas the lowest concentration c = 10 pM does not reach the equilibrium, even after 4 h injection time (see Figure 2A). 

Attenuated kinetic properties of the primary antibody resulted in dramatically increased time to equilibrium. The factor-10 lower association rate constant of *k_a_* = 1.0 × 10^5^ M^−1^ s^−1^ does not reach equilibrium after 4 h target injection, even with a concentration of c = 810 pM (see Figure 2B).

The expected target response, although injected over more than 60 min, is expected to be at the low end of the detection range. Thus, combined with baseline drifts and the presence of the complex media during the native target injection, a reliable quantification of the target-enrichment itself is prevented. 

To determine reliable kinetic data for the interaction of native targets and Fabs, an R_max_ of at least 5 RU should be achieved. 

## 4. Results and Discussion

### 4.1. In Silico-Determined Assay Settings Successfully Proven In Vitro

**Figure 3:**
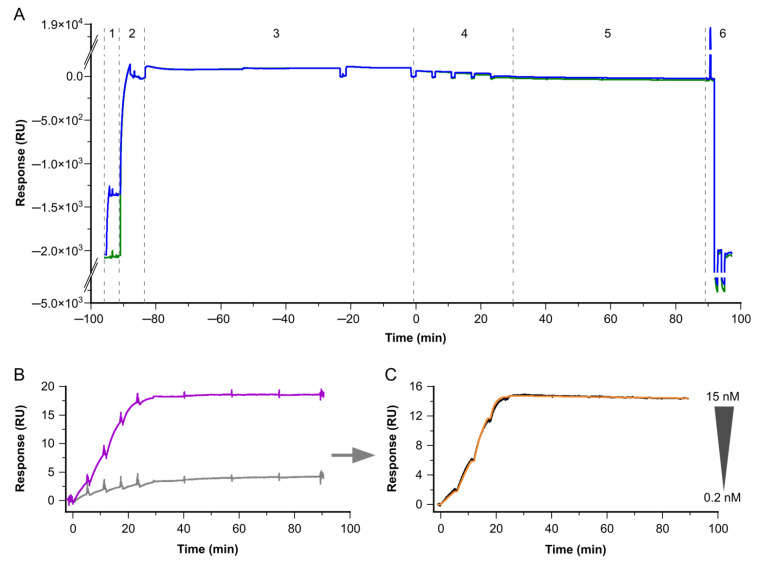
Exemplary sensorgrams for the determination of kinetic binding constants for low-abundant native targets and antibodies. The sensorgrams show the response signal in resonance units (RU) plotted against the time in minutes (min). (**A**) Sensorgrams for complete cycles of non-referenced data on Fc1 (reference, green) and Fc2 (active, blue). The different steps are depicted by the dotted lines. (1) The first antibody is bound on Fc2 by a covalently attached capture antibody. (2) Subsequently, a non-target-related antibody of the same species as the primary antibody is injected on Fc1 and 2. This antibody serves for referencing on Fc1, and additionally blocks free binding sites of the capture antibody on Fc1 and 2. (3) In the next step, the target is injected for t = 80 min. (4) Next, five consecutive injections with increasing concentrations of the second sandwich-pair antibody are applied, followed by (5) the dissociation phase. (6) Finally, the regeneration is applied. Here, the second binder represents an antibody Fab fragment, to ensure monovalent binding and to determine the interaction’s affinity. (**B**) Zoomed-in view of the second binder binding to the target as shown in (**A**); now, Fc2 is referenced versus Fc1, resulting in Fc2-1. The antibody concentration series (magenta) is overlaid with a blank, using a buffer instead of a second antibody (light gray). (**C**) Identical interaction to that shown in (**B**); here, double-referenced vs. blank (black) overlaid by the Langmuir 1:1 fitting (orange).

The simulated assay settings for the target enrichment were experimentally proven using the target-protein neurofilament light chain (NFL). NFL can only be found at very low concentrations in CSF samples from patients, and it is even lower concentrated in healthy subjects [10]. Therefore, the determination of binding kinetics with native NFL is a challenging task, and requires very long target-enrichment times, of up to t = 80 min. Based on this enrichment step, we were able, for the first time, to determine the kinetic rate constants and resulting affinity for Fab-fragments binding to surface-enriched, low-abundant, native NFL, from CSF (see Figure 3). 

Recently, Paiva et al. described a similar capturing approach with recombinant proteins from cellular extracts carrying an AviTag, which can directly bind to a Streptavidin SPR-chip after cellular biotinylation. Subsequently, the binding kinetics of the captured protein binding its target can be directly measured [21]. In 2012, an assay setup already demonstrated the capturing of antibodies directly from cell-culture supernatants utilizing appropriate immobilized species-specific capture antibodies. Afterwards the captured antibodies from supernatants were kinetically analyzed, in a screening-like fashion [22]. However, concentrations of overexpressed proteins are generally much higher than most protein markers in patient samples, and therefore require neither very-long enrichment times nor high-affine capture systems.

### 4.2. Proved Technical Reproducibility of Antibody Kinetics with Native GDF15 from Donors of Different Sources 

For growth differentiation factor 15 (GDF15), a disulfide-linked homodimer, the antibody recognition for binding native GDF15 from donors of different source material, was compared to recombinant GDF15. Human sera from a healthy donor, a patient with oncological disease, a donor with cardiovascular origin, and two pregnant donors served as native GDF15. The binding properties for monovalent binding anti-GDF15 Fab B, used as a second binder, were investigated. As a primary binder, Anti-GDF15 antibody A was used, with a fast association rate constant *k_a_* > 5.0 × 10^5^ M^−1^ s^−1^ and a superior GDF15-complex half-life time of t_/*2diss*_ > 231 min. 

**Figure 4:**
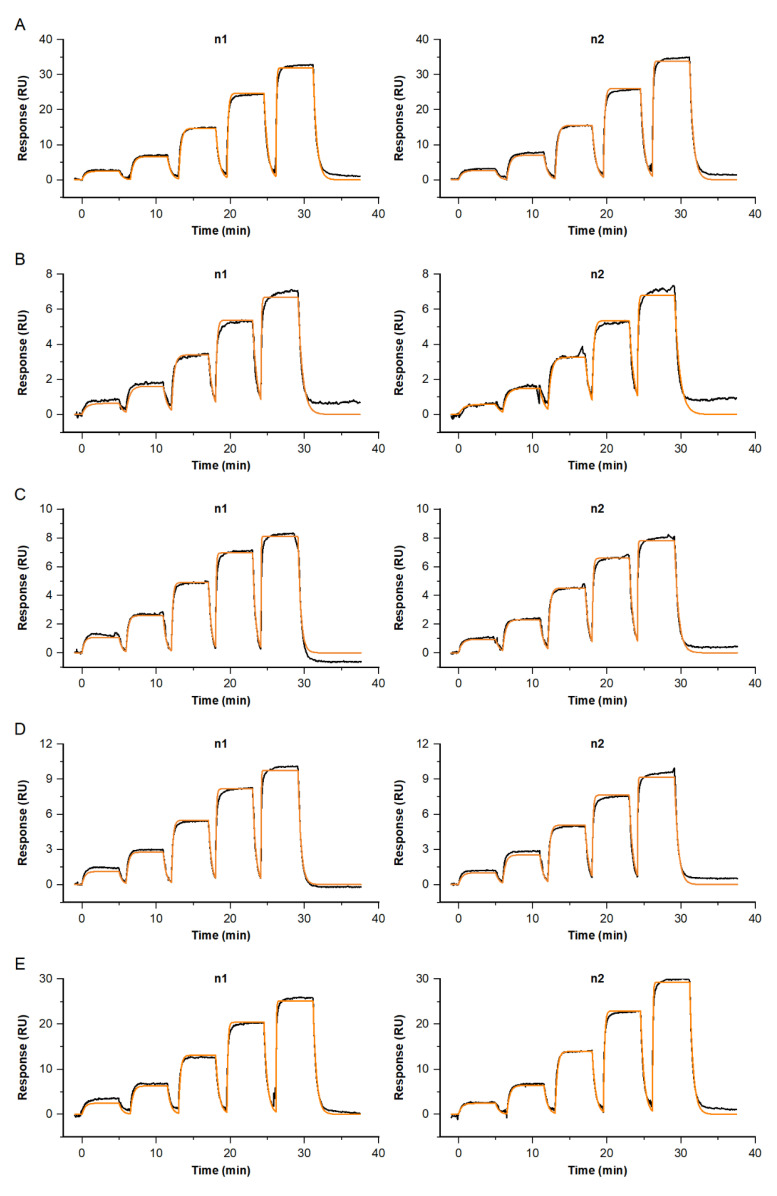
Kinetic profiles for Fab anti-GDF15 B binding to recombinant or native GDF15 of different sources. Shown are the double-referenced sensorgrams of the concentration series c = 7.4, 22.2, 66.7, 200 and 600 nM (black), overlaid by a Langmuir 1:1 fitting model, R_max_ global, RI = 0 (orange). The affinities for the GDF15 interactions were independently analyzed. Each sample was analyzed in duplicate. Binding to (**A**) recombinant GDF15, (**B**) native GDF15 derived from healthy donor, (**C**) native GDF15 derived from a patient with oncological disease, (**D**) native GDF15 derived from cardiovascular origin, (**E**) native GDF15 derived from pregnant donors.

All binding signatures for Fab B binding to native GDF15 of different origins—healthy, diseased or pregnant—how comparable fast on-/off profiles, not reaching saturation at the highest-tested Fab concentration of c = 600 nM (see Figure 4). The obtained *K_D_* represents the affinity, due to the monovalent binding of the Fab 2 to surface-enriched, primary antibody-bound GDF15. The affinities for all GDF15 samples, native and recombinant GDF15, were measured in duplicate, and ranged between *K_D_* = 78 and 124 nM (see Table 1).

The negative control for native GDF15 without the primary antibody shows no binding, and behaves in a buffer-like way (see Supplementary Section S2).

The Fab B shows comparable binding properties and affinities when binding to native GDF15 from patient samples of different sources. Here, it is shown for healthy, pregnant or oncological donors, and for cardiovascular origin. Furthermore, our results reveal no differences between the specific immune-complex formations with GDF15 from donor samples of the different sources. This is an initial indication that the targeted epitope regions in GDF15 are not altered under diseased conditions. 

### 4.3. Small Study Reveals Comparable Kinetic Constants for Native GDF15 from Different Sources

**Figure 5:**
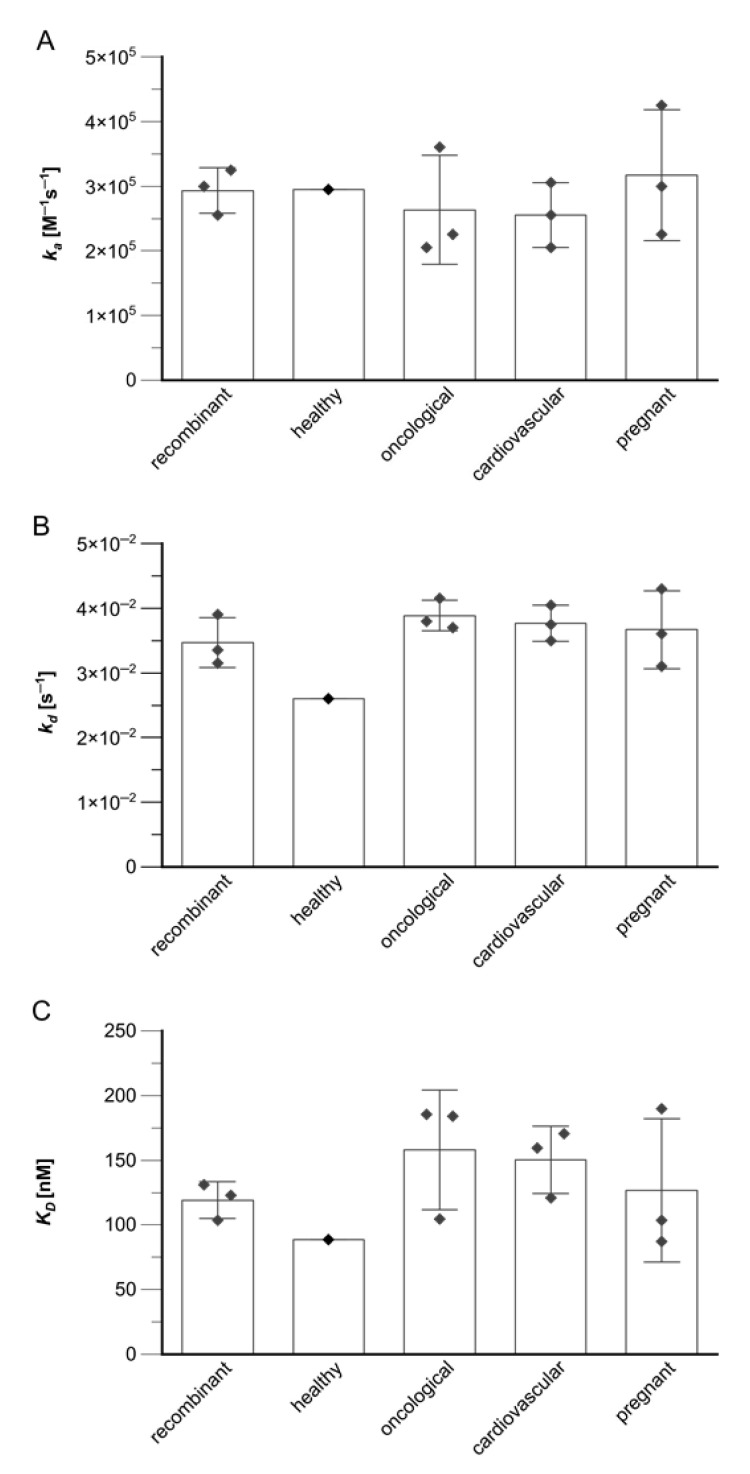
Comparison of kinetic constants for recombinant and native GDF15 derived from donors of certain sources. (**A**–**C**) Bars represent the means of the kinetic constants (donors: N = 3; except for healthy control, N = 1), while error bars depict the according standard deviations. In addition, the individual determined constants for each donor are represented as squares. Those donor constants were determined as technical duplicates. *k_a_*, *k_d_* and *K_D_* do not show significant differences among the different studies.

To further investigate the comparability among different sources, we started a small study to determine the kinetic constants for three donors (N = 3) from each source, except for the healthy control (N = 1). Each donor sample was measured as a technical duplicate, as described in Section 3.2. The resulting mean values for *k_a_*, *k_d_* and *K_D_* were compared, and underline our initial finding: the kinetic properties for GDF15 of the selected antibody-sandwich pair do not significantly differ throughout the tested donor samples from the different sources (see Figure 5, Supplementary Material Figure S2 and Table S2).

Based on our findings, this new approach will also be feasible for identifying antibody pairs, which can differentiate between healthy and diseased states. This can, for example, be realized by antibodies specific to a disease-related post-translational modification. Another opportunity for the diagnostic assay development is that, once the antibodies have been proven to have similar kinetic properties for intended-use samples and secondary material, the assay development can be streamlined. Often, the optimal intended-use samples for an assay development are very precious and hard to obtain; for example, when the number of patients is very low or when the use is ethically questionable. In those cases, it would be of great advantage if secondary material could be used, as it would allow for the speeding up of the diagnostic assay development, or even make it possible. One example of very precious material are CSF samples, which have been used in the following sections to identify antibody-sandwich pairs for native NFL.

### 4.4. Kinetic Constants for Antibodies Binding to Native Neurofilament Light Chain from Patients’ Cerebrospinal Fluid (CSF)

**Figure 6:**
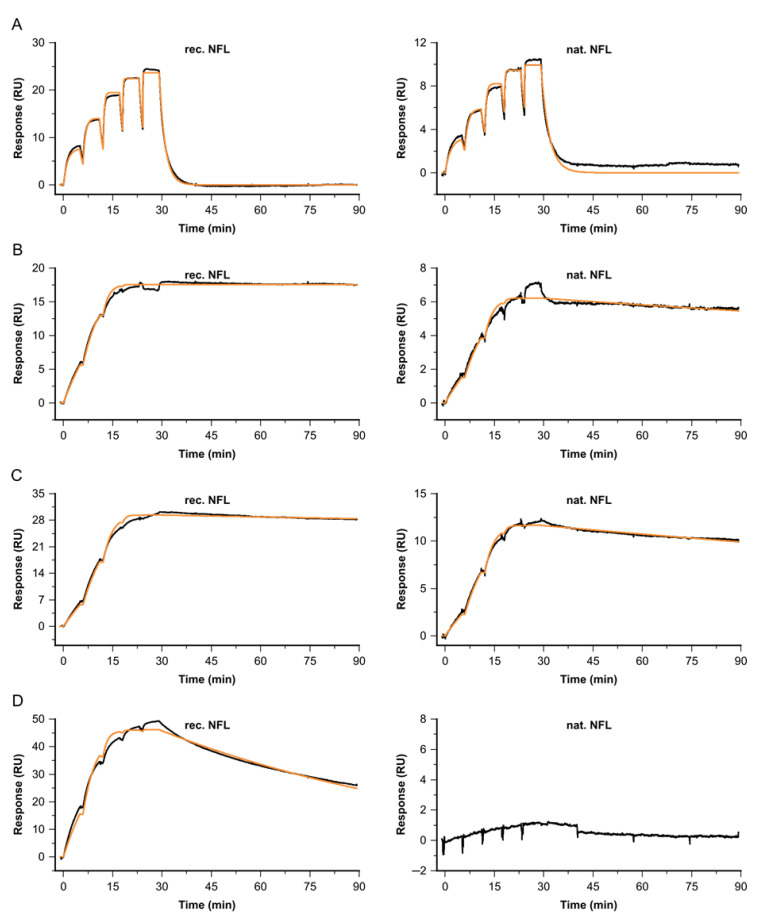
Kinetic profiles for a selection of Fabs binding to recombinant NFL (left) vs. native NFL from cerebrospinal fluid (right), at 37 °C. Sensorgrams (black) are overlaid with a Langmuir 1:1 fitting model, R_max_ global, RI = 0 (orange). Concentrations for (**A**) Fab A, (**B**) Fab B, (**C**) Fab C and (**D**) Fab I were varied, depending on their kinetic properties: c(A) = 3.7, 11.1, 33.3, 100, 300 nM, c(B) = 0.4, 1.1, 3.3, 10, 30 nM, c(C) = 0.2, 0.6, 1.7, 5, 15 nM and c(I) = 1.5, 4.4, 13.3, 40, 120 nM.

Comparable kinetic properties for binding native versus recombinant NFL were obtained for several Fabs (see Figure 6 and Table 2). Fab C shows superior kinetic properties, with affinities of *K_D_* = 2.8 pM for native and *K_D_* = 3.8 pM for recombinant NFL. For native and recombinant targets, the obtained complex half-life times at 37 °C were longer than *t_/2diss_* = 16 h. Fab A shows a binding signature with fast association and dissociation, resulting in an affinity of *K_D_ =* 8 nM and a complex half-life time below *t_/2diss_* = 1 min. Fab fragment B shows slightly complex binding behavior; therefore, the stated constants represent apparent values. Nevertheless, the complex half-life time for the interaction with native NFL is shortened, compared to recombinant NFL. Antibody I was identified as not binding to enriched native NFL. The negative control with buffer injections instead of the NFL behaved in a buffer-like way.

**Figure 7:**
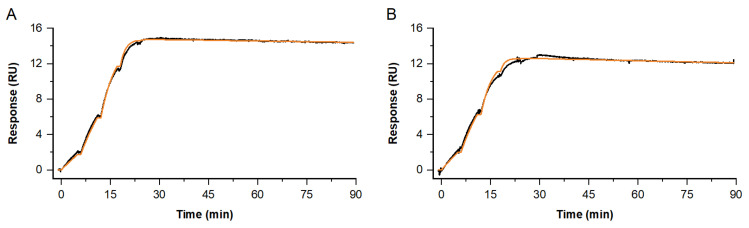
Single-cycle kinetics for the interaction of Antibody fab fragment C and native NFL at 37 °C. Depicted sensorgrams were obtained from two independent analyses: Fab C (c = 0.2, 0.6, 1.7, 5, 15 nM) binding to native NFL, with antibody A (**A**) or B (**B**) used as primary antibodies, both covering two different epitope regions. Sensorgrams (black) are overlaid with a Langmuir 1:1 fitting model, Rmax global, RI = 0 (orange).

Furthermore, the obtained kinetic profiles were proven to be reproducible in independent single-cycle experiments. Here, examples are shown for the antibody Fab fragment C interacting with native NFL (see Figure 7). In these experiments, two different primary antibodies were used to enrich NFL: antibody A or B. Those two are also representative antibodies, which bind in two different epitope regions on NFL. To compare the impact of avidity-burdened bivalent vs. monovalent binding on the complex half-life time, we investigated the binding of a Fab fragment and parental IgG (see Supplementary Material Figure S1 and Table S1).

### 4.5. Empirical Relation for Offered Amount of Native NFL and Its Surface Density

The total amount of the enriched, native NFL was calculated for the interaction of Fab C/native NFL, as shown in Figure 6C. Approx. 20 fmol native NFL is offered to the primary antibody during the 80 min enrichment, with an NFL concentration of approx. 25 pM and a flow rate of 10 µL/min. The achieved response of the native target is calculated using the formula:

R(target) = Rmax (secondary Ab) × M (target)/M (secondary Ab)(3)

Based on the molecular mass of NFL of *M =* 35.3 kDa and the experimental response maximum R_max_ = 13 RU for the interaction of Fab C and native NFL, the surface density is 9.2 RU. 

This translates into a surface density of NFL of approximately 0.2–0.3 fmol/mm^2^, based on the information (1000 RU = 1 ng/mm^2^) taken from S. Löfås et al. [23].

### 4.6. Identifying Antibodies Binding to Native NFL from CSF 

**Figure 8:**
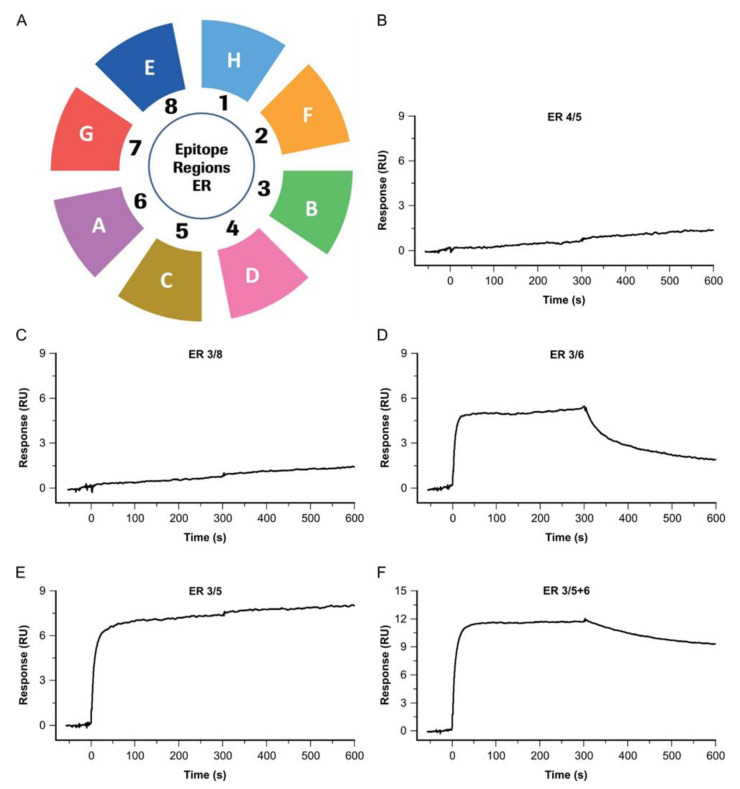
Bin chart and binding signatures of secondary antibodies binding to native NFL from CSF for five different antibody combinations. (**A**) So-called Bin chart of the SPR-based Epitope Binning experiments. Antibodies A, B, C, D E, F G and H cover eight distinct Epitope Regions (ER) on recombinant NFL. Based on the Epitope Binning results, antibody pairs have been selected to test for sandwich-complex formations with native samples. (**B**–**F**) Sensorgrams of the investigated Ab pairs binding native NFL. (**B**) Antibody pair D/C, covering Epitope regions (ER) 4/5; (**C**) Antibody pair B/E (ER 3/8); (**D**) Antibody pair B/A (ER 3/6); (**E**) Antibody pair B/C (ER 3/5; (**F**) Antibody-pair B/C and A (ER 3 and 5/6).

The identification of well-functioning antibody-sandwich pairs binding their native target is crucial in our diagnostic assay development, as well as for further kinetic analyses (see Section 4.1). To identify those pairs, antibodies from different epitope regions are combined and tested for their native-target recognition. Thereby, we can also identify and exclude, early on in the development process, those antibodies which cover epitope regions found in the recombinant but not the native target, as in the examples shown here, for NFL (see Figure 8).

The antibodies A–H cover eight distinct epitope regions on recombinant NFL, as listed below: A—ER6; B—ER3; C—ER5; D—ER4; E—ER8; F—ER2, G—ER7; H—ER1.

Antibodies representing different epitope regions form a sandwich complex with each other, whereas antibodies from an identical ER do not form a sandwich. In the case of overlapping epitopes in an epitope region, the so-called epitope bins, the order of the complex formation can matter, and affects whether a sandwich is formed or not.

A selection of antibody pairs were tested with native NFL, for the sandwich-complex formation: 

Antibody D (ER4) shows no sandwich-complex formation when combined with C (ER 5)—see overlay (A). As antibody C (ER 5) forms a sandwich when combined with antibody B (ER 3)—see overlay (D), antibody D is identified as not binding, or weakly binding, to native NFL, despite the high-affine binding of recombinant NFL. Accordingly, E—representing ER 8—is identified as not, or too-weakly, binding to native NFL—see overlay (B). Antibodies covering the three ERs 3, 5, and 6 show preferable binding to native NFL. When combining C and A as secondary antibodies, using B as a primary antibody, a signal amplification for binding NFL from CSF is achieved—see overlay (E).

## 5. Conclusions

Our results clearly demonstrate that the here-described SPR-based method enables the determination of kinetic properties for the interaction of antibodies binding their native targets from human CSF, sera or plasma. Through this approach, affinities in the low 3-digit-nM to 1-digit-pM range could be measured. 

Our assay set-up overcomes the limitation of detection for low concentrated native antigens from patients in the pM-range, by using the following approach: instead of the conventional assay setup with a captured, surface-displayed antibody as ligand and the antigen as analyte in solution, a sandwich-assay format is used, to enrich the native target sufficiently. There is no need to enrich and purify the native target before it is analyzed via SPR, thus lowering the risk of protein alterations due to purification conditions. 

At the same time, we overcome the challenges of measuring kinetic properties in the presence of complex matrices like body fluids. However, it must be mentioned that native samples can still be challenging, as the complex matrices of body fluids can lead to non-specific binding or aggregation, and make kinetic investigations impossible. This is of special importance when the marker is not naturally occurring in the given body fluid but is rather released, due to disease conditions. Therefore, SPR-assay optimizations can become necessary, but at the same time such results also give indications about the feasibility of the final diagnostic assay. 

The proposed SPR assay was used to identify antibodies binding the recombinant, but not the native target. This is of great interest in the binder development, to exclude candidates early on which otherwise would have been processed further. To do so in an efficient way, the needed requirements are high, especially if kinetics for the native marker should also be determined: not only are several sandwich pairs of at least two high-affinity antibodies binding to different epitope regions required, but also the ability to generate Fab fragments of those antibodies.

The measurement of binding properties for recombinant vs. native targets is of great value, as such comparisons generate insights into construct differences like epitope accessibility. The native folding and native co-interactions may differ from the recombinant constructs, and therefore matter during diagnostic assay or pharmaceutical drug development. Based on these data, conclusions can be drawn regarding the native soluble targets’ character; for example, post-translational modifications or isoforms, which bring the research and development process one step closer to the patient.

Our SPR-assay setup can also help to speed and ease up diagnostic assay or pharmaceutical drug development, especially when comparable kinetic properties for intended use and secondary material can be proven. Those results would then allow for secondary-material usage during assay or drug development, which becomes of special interest when intended-use samples are precious, hard to obtain, or ethically questionable.

Shortening development timelines for diagnostic assays, and likewise reducing the attrition rates for the development of pharmaceutical drugs, is of utmost importance for the patients and our future health-care systems.

## Figures and Tables

**Table 1:** Kinetic constants and affinities for native GDF15 from patient samples vs. recombinant GDF15 binding Fab Anti-GDF15 B. SE—Standard error of mean of the SPR fit in comparison to the measured data points.

GDF15 Origin	*k_a_* [M^−1^ s^−1^]	SE (*k_a_*) [%]	*k_d_* [s^−1^]	SE (*k_d_*) [%]	*t*_/*2diss*_ [min]	*K_D_* [nM]	Rmax [RU]	U-Value
recombinant	3.3 × 10^5^	0.8	3.4 × 10^−2^	0.6	<1	103	37.4	4
recombinant	3.2 × 10^5^	0.9	3.3 × 10^−2^	0.7	<1	104	39.7	4
native, healthy	3.3 × 10^5^	2.3	2.8 × 10^−2^	1.9	<1	83	7.6	12
native, healthy	2.6 × 10^5^	3.2	2.4 × 10^−2^	2.5	<1	94	7.9	15
native, oncological	4.4 × 10^5^	2.0	4.3 × 10^−2^	1.6	<1	98	8.8	7
native, oncological	2.8 × 10^5^	1.3	3.1 × 10^−2^	1.1	<1	111	8.6	5
native, cardiovascular	4.8 × 10^5^	0.7	3.7 × 10^−2^	0.6	<1	78	28.4	3
native, cardiovascular	3.7 × 10^5^	1.3	3.5 × 10^−2^	1.2	<1	96	33.9	4
native, pregnant	4.8 × 10^5^	0.7	3.7 × 10^−2^	0.6	<1	78	28.4	3
native, pregnant	3.7 × 10^5^	1.3	3.5 × 10^−2^	1.2	<1	96	33.9	4

**Table 2:** Kinetic constants and affinities for a selection of antibody Fab fragments binding to recombinant NFL vs. native NFL, from CSF at 37 °C. SE—Standard error of mean of the SPR fit in comparison to the measured data points.

2nd Fab	1st Ab	Biomarker	*k_a_* [M^−1^ s^−1^]	±SE (*k_a_*) [M^−1^ s^−1^]	*k_d_* [s^−1^]	±SE (*k_d_*) [s^−1^]	*t*_/*2diss*_ [min]	*K_D_* [M]	Rmax [RU]	U-Value
A	B	recombinant NFL	1.2 × 10^6^	3.9 × 10^3^	9.2 × 10^−3^	2.5 × 10^−5^	1	8.0 × 10^−9^	24	2
A	B	native NFL from CSF	8.4 × 10^5^	1.0 × 10^4^	6.8 × 10^−3^	6.5 × 10^−5^	2	8.0 × 10^−9^	10	7
B	J	recombinant NFL	3.2 × 10^6^	6.9 × 10^3^	1.0 × 10^−5^	2.8 × 10^−8^	1155	3.2 × 10^−12^	18	95
B	J	native NFL from CSF	2.3 × 10^6^	1.2 × 10^4^	3.6 × 10^−5^	5.3 × 10^−7^	322	1.5 × 10^−11^	6	5
C	B	recombinant NFL	3.6 × 10^6^	9.7 × 10^3^	1.0 × 10^−5^	2.7 × 10^−7^	1155	2.8 × 10^−12^	29	12
C	B	native NFL from CSF	2.9 × 10^6^	1.1 × 10^4^	1.1 × 10^−5^	2.4 × 10^−7^	1058	3.8 × 10^−12^	13	9
C	A	native NFL from CSF	2.1 × 10^6^	2.9 × 10^3^	1.0 × 10^−5^	1.5 × 10^−7^	1155	4.7 × 10^−12^	15	9
I	B	recombinant NFL	8.4 × 10^5^	3.8 × 10^3^	1.7 × 10^−4^	5.1 × 10^−7^	67	1.8 × 10^−10^	46	2
I	B	native NFL from CSF	n.b.		n.b.		n.b.	n.b.	n.b.	n.b.

## Data Availability

The data presented in this study are available on request from the first author.

## Supplementary Materials

The following supporting information can be downloaded at: https://www.mdpi.com/article/10.3390/bios13121030/s1, Figure S1: Single-cycle kinetics for surface displayed sTREM-1 binding to Fab and its parental IgG antibody at 25 °C; Table S1: Kinetic constants and affinities or avidities for a Fab and IgG antibody binding to surface-displayed sTREM-1 of different sources; Figure S2: Exemplary negative control for 2nd binder; Table S2: Kinetic constants for small study of native GDF15 from different sources.

## References

[1] Mahmuda A., Bande F., Abdulhaleem N., Al-Zihiry K.J.K., Majid R.A., Hamat R.A., Abdullah W.O., Zasmy N. (2017). Monoclonal antibodies in immunodiagnostic assays: A review of recent applications, Sokoto. J. Vet. Sci..

[2] Fesseha H. (2020). Monoclonal Antibody and its Diagnostic Application—Review. Biomed. J. Sci. Tech. Res..

[3] Siddiqui M.Z. (2010). Monoclonal Antibodies as Diagnostics; an Appraisal. Indian J. Pharm. Sci..

[4] Shakya R., Nguyen T.H., Waterhouse N., Khanna R. (2020). Immune contexture analysis in immuno-oncology: Applications and challenges of multiplex fluorescent immunohistochemistry. Clin. Transl. Immunol..

[5] Schindler S.E., Gray J.D., Gordon B.A., Xiong C., Batrla-Utermann R., Quan M., Wahl S., Benzinger T.L.S., Holtzman D.M., Morris J.C. (2018). Cerebrospinal fluid biomarkers measured by Elecsys assays compared to amyloid imaging. Alzheimer’s Dement..

[6] Assadi A., Zahabi A., Hart R.A. (2020). GDF15, an update of the physiological and pathological roles it plays: A review, Pflügers. Arch. Eur. J. Physiol..

[7] Delaby C., Bousiges O., Bouvier D., Fillée C., Fourier A., Mondésert E., Nezry N., Omar S., Quadrio I., Rucheton B. (2022). Neurofilaments contribution in clinic: State of the art. Front. Aging Neurosci..

[8] Gaetani L., Blennow K., Calabresi P., Filippo M.D., Parnetti L., Zetterberg H. (2019). Neurofilament light chain as a biomarker in neurological disorders. J. Neurol. Neurosurg. Psychiatry.

[9] Agrawal N., Farhat N.Y., Sinaii N., Do A.D., Xiao C., Berry-Kravis E., Bianconi S., Masvekar R., Bielekova B., Solomon B. (2022). Neurofilament light chain in cerebrospinal fluid as a novel biomarker in evaluating both clinical severity and therapeutic response in Niemann-Pick disease type C1. Genet. Med. Off. J. Am. Coll. Méd. Genet..

[10] Arslan B., Zetterberg H. (2023). Neurofilament light chain as neuronal injury marker—What is needed to facilitate implementation in clinical laboratory practice?. Clin. Chem. Lab. Med. (CCLM).

[11] Rezabakhsh A., Rahbarghazi R., Fathi F. (2020). Surface plasmon resonance biosensors for detection of Alzheimer’s biomarkers; an effective step in early and accurate diagnosis. Biosens. Bioelectron..

[12] Xia N., Liu L., Harrington M.G., Wang J., Zhou F. (2010). Regenerable and Simultaneous Surface Plasmon Resonance Detection of Aβ(1−40) and Aβ(1−42) Peptides in Cerebrospinal Fluids with Signal Amplification by Streptavidin Conjugated to an N-Terminus-Specific Antibody. Anal. Chem..

[13] Lisi S., Scarano S., Fedeli S., Pascale E., Cicchi S., Ravelet C., Peyrin E., Minunni M. (2017). Toward sensitive immuno-based detection of tau protein by surface plasmon resonance coupled to carbon nanostructures as signal amplifiers. Biosens. Bioelectron..

[14] Špringer T., Hemmerová E., Finocchiaro G., Krištofiková Z., Vyhnálek M., Homola J. (2020). Surface plasmon resonance biosensor for the detection of tau-amyloid β complex. Sens. Actuators B Chem..

[15] Kim S., Lee H.J. (2015). Direct Detection of α-1 Antitrypsin in Serum Samples using Surface Plasmon Resonance with a New Aptamer–Antibody Sandwich Assay. Anal. Chem..

[16] Kim S., Wark A.W., Lee H.J. (2016). Femtomolar Detection of Tau Proteins in Undiluted Plasma Using Surface Plasmon Resonance. Anal. Chem..

[17] Kim S., Lee H.J. (2017). Gold Nanostar Enhanced Surface Plasmon Resonance Detection of an Antibiotic at Attomolar Concentrations via an Aptamer-Antibody Sandwich Assay. Anal. Chem..

[18] Karlsson R., Katsamba P.S., Nordin H., Pol E., Myszka D.G. (2006). Analyzing a kinetic titration series using affinity biosensors. Anal. Biochem..

[19] Gaetani L., Höglund K., Parnetti L., Pujol-Calderon F., Becker B., Eusebi P., Sarchielli P., Calabresi P., Filippo M.D., Zetterberg H. (2018). A new enzyme-linked immunosorbent assay for neurofilament light in cerebrospinal fluid: Analytical validation and clinical evaluation. Alzheimer’s Res. Ther..

[20] Karlsson R., Fridh V., Frostell Å. (2018). Surrogate potency assays: Comparison of binding profiles complements dose response curves for unambiguous assessment of relative potencies. J. Pharm. Anal..

[21] Paiva A.C.F., Lemos A.R., Busse P., Martins M.T., Silva D.O., Freitas M.C., Santos S.P., Freire F., Barrey E.J., Manival X. (2023). Extract2Chip—Bypassing Protein Purification in Drug Discovery Using Surface Plasmon Resonance. Biosensors.

[22] Schräml M., Biehl M. (2012). Kinetic screening in the antibody development process. Methods Mol. Biol..

[23] Löfås S., Lagerström K. (1998). Solid Phase Binding. Assay. Patent.

