# Supplementary material for: Antibody Profiling: Kinetics with Native Biomarkers for Diagnostic Assay and Drug Developments
Source: Biosensors (Basel). 2023 Dec 14;13(12):1030. doi: 10.3390/bios13121030 (PMC10742008; doi:10.3390/bios13121030)
Supplement: Supplementary file 1 [file biosensors-13-01030-s001.zip › biosensors-2701471-supplementary.pdf]

biosensors

MDPI

Supplementary Materials

Antibody Profiling: Kinetics with Native Biomarkers for Diagnostic Assay and Drug Developments

Ute Jucknischke *, Sebastian Friebe, Markus Rehle, Laura Quast and Sven H. Schmidt *

Diagnostic Solutions, Reagent Research and Design, Department Antibody and Protein Technologies, (DSRRA) at Roche Diagnostics GmbH, Nonnenwald 2, 82377 Penzberg, Germany

* Correspondence: ute.jucknischke@roche.com (U.J.); sven_h.schmidt@roche.com (S.H.S.)

S.1 Affinity vs. avidity

Triggering receptor expressed on myeloid cells-1 is a transmembrane receptor expressed by innate immune cells, including endothelial cells and platelets. Its released soluble factor sTREM-1 serves as a biomarker related to sepsis.

We compared the impact of avidity-burdened bivalent vs. monovalent binding on the complex half-life time for antibody Anti-sTREM-1 binding to surface-displayed sTREM-1, using a Fab and the IgG-format of the parental antibody as 2nd binder. With the stated assay protocol, see Section 2, we used a primary antibody with a fast association rate constant k_a > 2.0 x 105 M─1s─1 and a sufficiently high complex half-life time >115 min, measured at 37°C.

The kinetic profiles for monovalent binding Fab show a detectable dissociation with complex-half-life times t/2 diss between 26–47 min, when binding to recombinant, native or LPS-stimulated sTREM-1, see Figure S1 and Table S1. The interactions obey the Langmuir law. No significant difference occurred for the analyzed differing sTREM-1 sources, recombinant, native or LPS-stimulated sTREM-1. The avidity-burdened binding of the bivalent binding IgG shows a significantly slowed down dissociation with half-life times t/2 diss > 460 min. Interestingly, the interaction shows a complex binding behavior, not observed for the monovalent Fab- binding. Therefore, only the dissociation phase was evaluated using the Langmuir dissociation fit. Approximately factor 2 deviating sTREM-1 densities, indicated by the achieved maximum binding response of the 2nd binder Rmax between 17 RU and 42 RU revals high enough density to enable bivalent binding to neighbored sTREM-1.

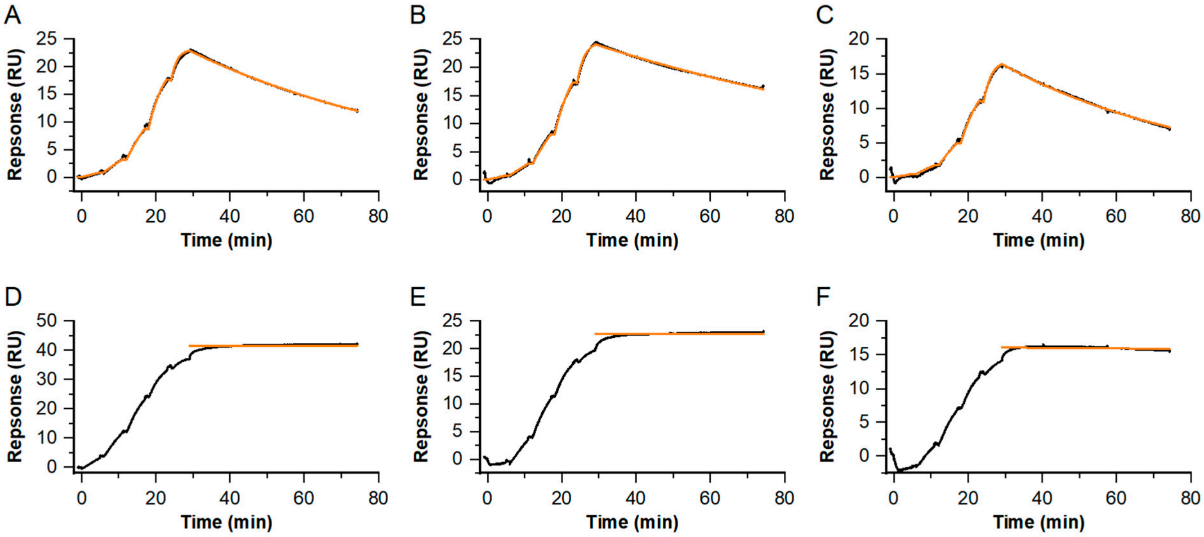

Figure S1. Single cycle kinetics for surface displayed sTREM-1 binding to Fab and its parental IgG antibody at 25°C; A series of five consecutive injections of the Fab or IgG Antibody was applied with concentration c= 1.5, 4.4, 13.3, 40, 120 nM; (A)–(C) Binding signatures for Fab binding to (A) recombinant sTREM-1 (B) native sTREM-1 from indented use-sudy (C) LPS-stimulated sTREM1 (black), with overlaid Langmuir 1:1 Fit (orange); (D)–(F) Binding signatures for parental IgG-antibody binding to (D) recombinant sTREM-1 (E) native sTREM-1 from indented use-study (F) LPS-stimulated sTREM1, depicted black, overlaid with the dissociation Fit (orange).

Table S1. Kinetic constants and affinities or avidities for a Fab and IgG Antibody binding to surface-displayed sTREM-1 of different sources. SE - Standard error of mean of the SPR-fit in comparison to the measured data points.

2nd binder	sTREM-1 origin	k_a [M⁻¹s⁻¹]	± SE(k_a) [M⁻¹s⁻¹]	k_d [s⁻¹]	± SE(k_d) [s⁻¹]	t/2 diss [min]	K_D [M]	Rmax [RU]	U-Value
Fab	recombinant	8.8×10^4	2.4×10^2	2.5×10^{-4}	$<1.0 \times 10^{-5}$	47	2.8×10^{-9}	23	1
Fab	native	5.9×10^4	5.4×10^2	4.5×10^{-4}	$<1.0 \times 10^{-5}$	26	7.6×10^{-9}	15	37
Fab	native - LPS-stimulated	6.3×10^4	1.3×10^2	3.0×10^{-4}	$<1.0 \times 10^{-5}$	38	4.8×10^{-9}	18	1
IgG-antibody	recombinant	n.d.	n.d.	$<2.5 \times 10^{-5}$	$<1.0 \times 10^{-5}$	>460	n.d.	44	n.d.
IgG-antibody	native	n.d.	n.d.	$<2.5 \times 10^{-5}$	$<1.0 \times 10^{-5}$	>460	n.d.	22	n.d.
IgG-antibody	native - LPS-stimulated	n.d.	n.d.	$<2.5 \times 10^{-5}$	$<1.0 \times 10^{-5}$	>460	n.d.	16	n.d.

S.2 Negative controls for Single Cycle Kinetics

During the experiments negative controls were analyzed, when determining the kinetic properties for antibodies binding to native, soluble biomarkers GDF15, NFL or sTREM-1.

Instead of the target-enrichment, buffer injections were performed, thus enabling the monitoring of possible non-specific binding of the 2nd binder to the primary antibody or the sensor-surface.

Additionally, the negative controls without a primary antibody present were monitored, subsequently to the native target-injection.

No significant binding is detectable, when, for example, Anti-GDF15-Fab B is injected as 2nd binder, subsequently to the native GDF15-injections without a primary antibody being captured via the surface-displayed capture system, see Figure S2.

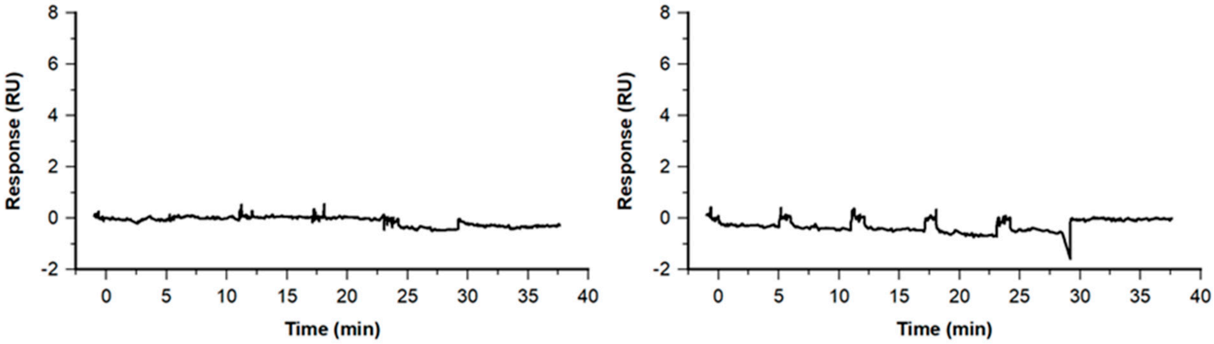

Figure S2. The negative control for 2nd binder Anti-GDF15 Fab native GDF15 without the primary antibody shows no binding and behaves buffer-like.

The controls with buffer injections instead of the 2nd binder, subsequently to the native target-enrichment via the primary antibody were used as blanks for the double-referencing as stated, see section 4.1 Fig 3.

S3 Small study binding constants

To further investigate the comparability between different sources of GDF15 we started a small study to determine the kinetic constants for three donors (N=3) from each source except for the healthy control (N=1). Each donor sample was measured as a technical duplicate. The resulting mean-values for ka, kd and KD were compared and can be found in Table S2.

Table S2. Kinetic constants for small study of native GDF15 from different sources, s. Figure 5. Section 4.3; Kinetic constants represent means of N = 3 donors; except for healthy control, N=1. Individual donor constants were determined as technical duplicates. SD - represents Standard deviation.

GDF15 Origin	k_a [$M^{-1}s^{-1}$]	± SD(k_a) [$M^{-1}s^{-1}$]	k_d [s^{-1}]	± SD(k_d) [s^{-1}]	K_D [nM]	± SD (K_D) [nM]
recombinant	2.9×10^5	2.9×10^4	3.5×10^{-02}	3.2×10^{-03}	119	12
healthy	3.0×10^5	-	2.6×10^{-02}	-	89	-
oncological	2.6×10^5	6.9×10^4	3.9×10^{-02}	1.9×10^{-03}	158	38
cardiovascular	2.6×10^5	4.1×10^4	3.8×10^{-02}	2.2×10^{-03}	150	21
pregnant	3.2×10^5	8.2×10^4	3.7×10^{-02}	4.9×10^{-03}	127	45
